# Subtle perturbations of ovarian steroidogenesis in patients classified as Poseidon Group 3. Which consequences for therapeutic strategy?

**DOI:** 10.3389/fendo.2024.1231585

**Published:** 2024-02-07

**Authors:** Jean Noel Hugues

**Affiliations:** University Sorbonne Paris Nord, Villetaneuse, France

**Keywords:** ovarian physiology, androgen -, anti-Mullerian hormone (AMH), folliculogenesis, steroid biosynthesis

## Abstract

The optimal strategy for stimulation of young women with a low ovarian reserve is still a challenging issue because the physio-pathogeny of this disorder is often unknown. As androgen production by the ovary plays a crucial role in folliculogenesis, it was tempting to speculate that subtle perturbations in ovarian steroidogenesis might participate to the low responsiveness to gonadotrophins. Indeed, *in vitro* analysis of human luteinized granulosa cells has recently provided evidence for some enzymatic deficits in steroidogenesis and altered response to gonadotrophins. Therefore, improving androgen environment of women classified in Poseidon Group 3 should be considered. In this clinical situation, the potential benefit of androgen supplementation or stimulation of theca cells by LH-activity products are respectively discussed.

## Introduction

A new stratification of low responders to ovarian stimulation has been recently described based on the combination of both qualitative (age) and quantitative parameters (serum AMH, Antral Follicular Count) allowing to identify 4 different groups (1). A hypo-response is usually associated with a poor prognosis of fertility when ovarian parameters are altered (AFC < 5 – AMH < 1.2 ng/ml), even in young (< 35 yrs) women (Poseidon Group 3). As physio-pathogeny of ovarian hypo-response is still often unknown and likely related to various mechanisms, strategies usually suggested such as prescription of higher FSH doses and/or addition of products with LH-activity proved to be partly ineffective to improve follicular recruitment and/or the number of good quality embryos.

It was well demonstrated that follicular development is dependent on several ovarian paracrine factors with a special focus on the critical role of androgens on ovarian physiology. The purpose of this article is to gain an insight into the consequences of an altered steroidogenesis on the ovarian response to stimulation and to discuss the optimal way to counteract this defect.

## Androgens and folliculogenesis

While androgens have traditionally been considered detrimental to ovarian function, the development of different types of androgen receptor (AR) knock out models along with various *in vivo* and invitro studies allowed to establish that androgen action through specific receptors is required to get normal ovarian development. In many species, AR are expressed in different ovarian compartments but are most abundant in the pre-antral/antral stages of follicular development while they decline as follicle matures to the pre-ovulatory stage (2). In primates, Weil et al. demonstrated that testosterone, in a positive feedback loop, increases AR expression in theca and granulosa cells of pre-antral follicles (3).

In 1981, Hillier et al. provided evidence that granulosa cell aromatase induction/activation by hFSH is an androgen receptor-regulated process (4). Regarding follicular development, several studies demonstrated that androgen treatment enhances pre-antral follicular growth and improves ovarian response by increasing granulosa cell proliferation (Ki67) and by attenuating follicular atresia (5). Furthermore, administration of androgens in monkeys initiates follicular recruitment, stimulation of early stages of follicular growth and increase in the number of growing follicles (6). While these observed effects could be blocked by anti-androgens (7), it is still uncertain whether they are the consequence of direct androgen actions or due to the conversion of androgens to oestrogens.

What about androgen signalling in the ovary? The classic mode of androgen action, as for most steroids, involves binding of androgen to AR in the cell cytoplasm and translocation of the hormone-receptor complex to the nucleus where it binds to a specific sequence in the promotor of the relevant target gene and promotes gene transcription. According to this model, androgens induce follicle-stimulating hormone receptor (FSHR) mRNA expression during pre-antral to antral follicle progression in different species including primates (6, 8). More recently, in human granulosa cells, a positive correlation has been found between mRNA levels of AR/FSHR and androgen levels in follicular fluid (9). Altogether, these data indicate that androgen stimulation enhances follicular sensitivity by increasing FSHR levels, which contributes to follicle growth. In addition, the study by Sen et al. (10) indicated that androgens increase FSHR protein levels in a nongenomic transcription-independent fashion. Indeed, androgens, through PXN-regulated genomic (Paxillin implicated in translocation of AR to the nucleus) as well as non-genomic AR actions, induce the expression of a micro-RNA (*miR-125b*) that decreases pro- apoptotic proteins, thereby contributing to androgen-induced follicular survival.

**Figure 1:**
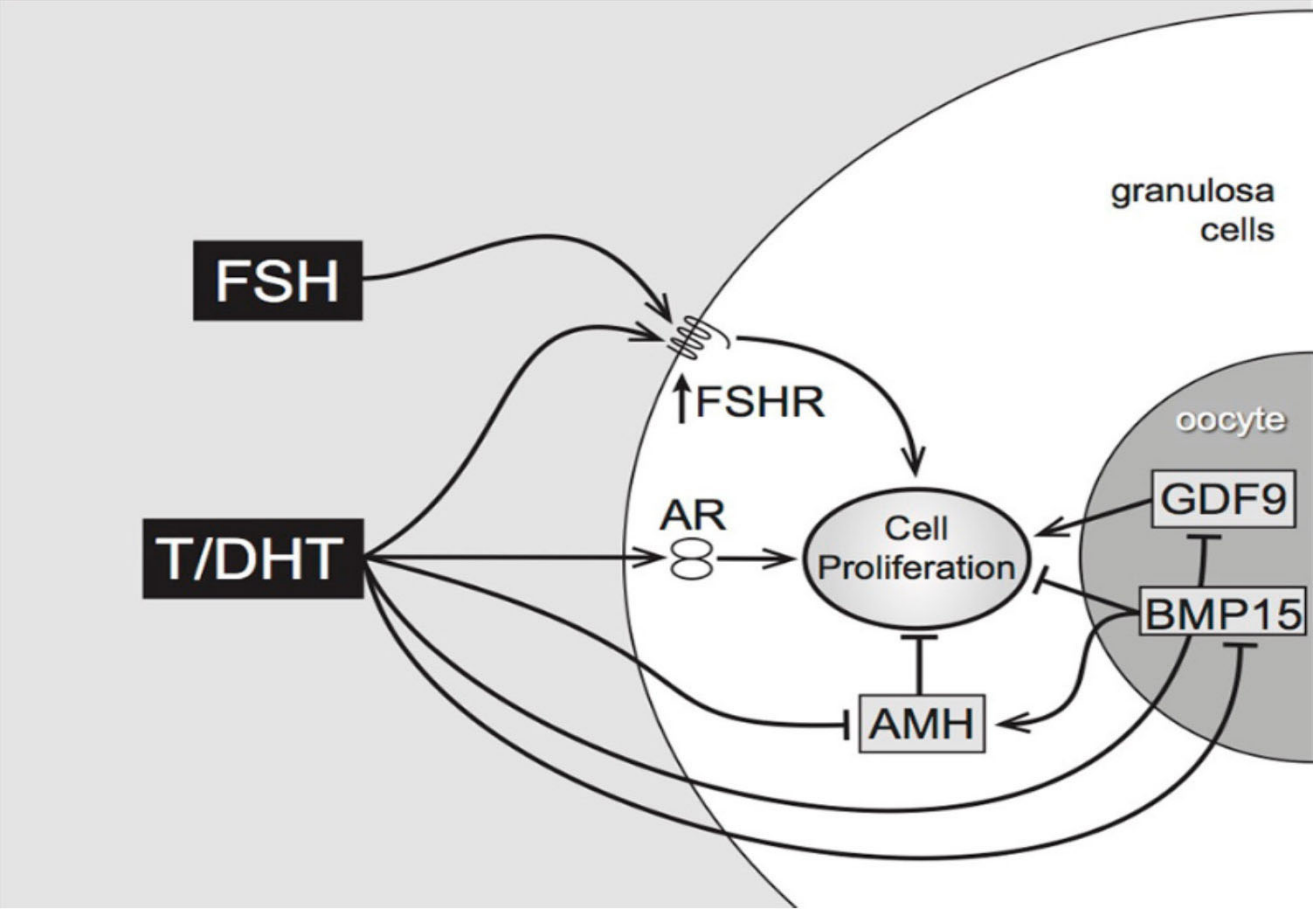
Proposed pathways of androgen action on preantral follicle growth (12). Testosterone or/and DHT act via the androgen receptor, increasing granulosa cell proliferation. This may be mediated directly, or indirectly by increased FSHR (stimulating GC proliferation) or decreased AMH (reducing AMH inhibition). AMH can be further reduced by androgen-induced reduction of oocyte-specific BMP15, which normally stimulates AMH levels.

Additionally, in isolated mouse preantral follicles, incubation with DHT greatly enhances expression of the steroid acute regulatory protein (StAR, a key regulator of steroidogenesis) in response to FSH (11). In the same model, both testosterone and DHT interact with members of the TGFβ superfamily, reducing gene expression of anti-Mullerian hormone (AMH) (produced by granulosa cells) and of bone morphogenetic protein- 15 (BMP-15) (produced by the oocyte) which may have inhibitory effects on follicle growth (11). As shown in **Figure 1**, the combined effects of DHT, positive on FSHR expression and negative on the growth- inhibitory AMH and BMP-15, indicate that the regulation of pre-antral follicular growth is dependent on a complex balance between endocrine and local growth factor actions (12).

Finally, Testosterone and DHT raise the transcript levels of insulin-like-growth 1 (IGF-1) and IGF1 receptors in primate ovaries (13). The presence of IGF- 1 and IGF-1 receptor mRNA in granulosa cells, theca, oocytes and interstitial cells and the fact that IGF-1 suppresses follicular apoptosis suggests that androgens may have a direct impact on improving the quality of oocytes and produced embryos (14).

Altogether these data clearly demonstrate the crucial role of androgens in the regulation of folliculogenesis. Therefore, we may speculate that any defect in androgen production could actually be associated with a hypo-response to stimulation. Consequently, a deep assessment of ovarian androgen biosynthesis could be valuable in patients with a poor ovarian response to FSH stimulation.

## Regulation of ovarian steroidogenesis

**Figure 2:**
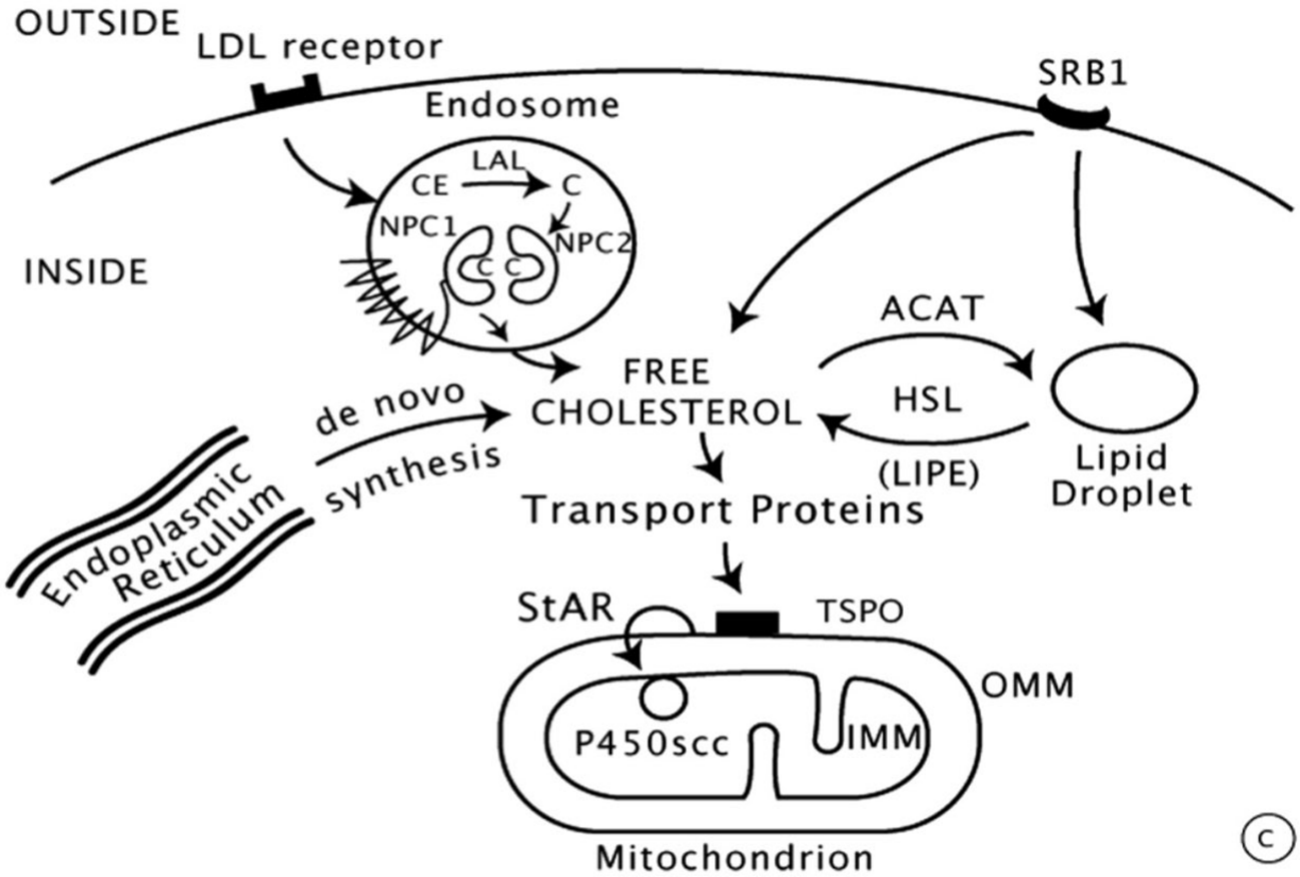
Intracellular cholesterol trafficking (8).

Steroid biosynthesis requires complex interactions that have been extensively studied. Cholesterol is the unique precursor derived from circulating LDL lipoproteins which are internalized by receptor-mediated endocytosis. LDL proteins are then degraded by proteolysis with liberation of cholesterol esters, subsequently hydrolyzed to free cholesterol by lysosomal acid lipase (LAL) (**Figure 2**) (15).

Then, the entry of cholesterol into steroidogenic mitochondria is dependent on action of StAR (Steroidogenic Acute Regulatory protein) which triggers the acute steroidogenic response. As shown in **Figure 2**, StAR acts on the outer mitochondrial membrane (OMM) and triggers cholesterol flux to inner mitochondrial membrane (IMM) where it can be converted to pregnenolone by 450scc to initiate steroidogenesis.

Most enzymes involved in steroid biosynthesis are either cytochrome P450s (CYPs) or Hydroxysteroid Dehydrogenases (HSDs). The human genome includes genes for 57 CYPs, 7 being targeted to the mitochondria (Type 1), the other 50 (Type 2) to the Endoplasmic Reticulum (ER). Six P450 enzymes participate in adrenal/ovarian steroidogenesis.

**Figure 3:**
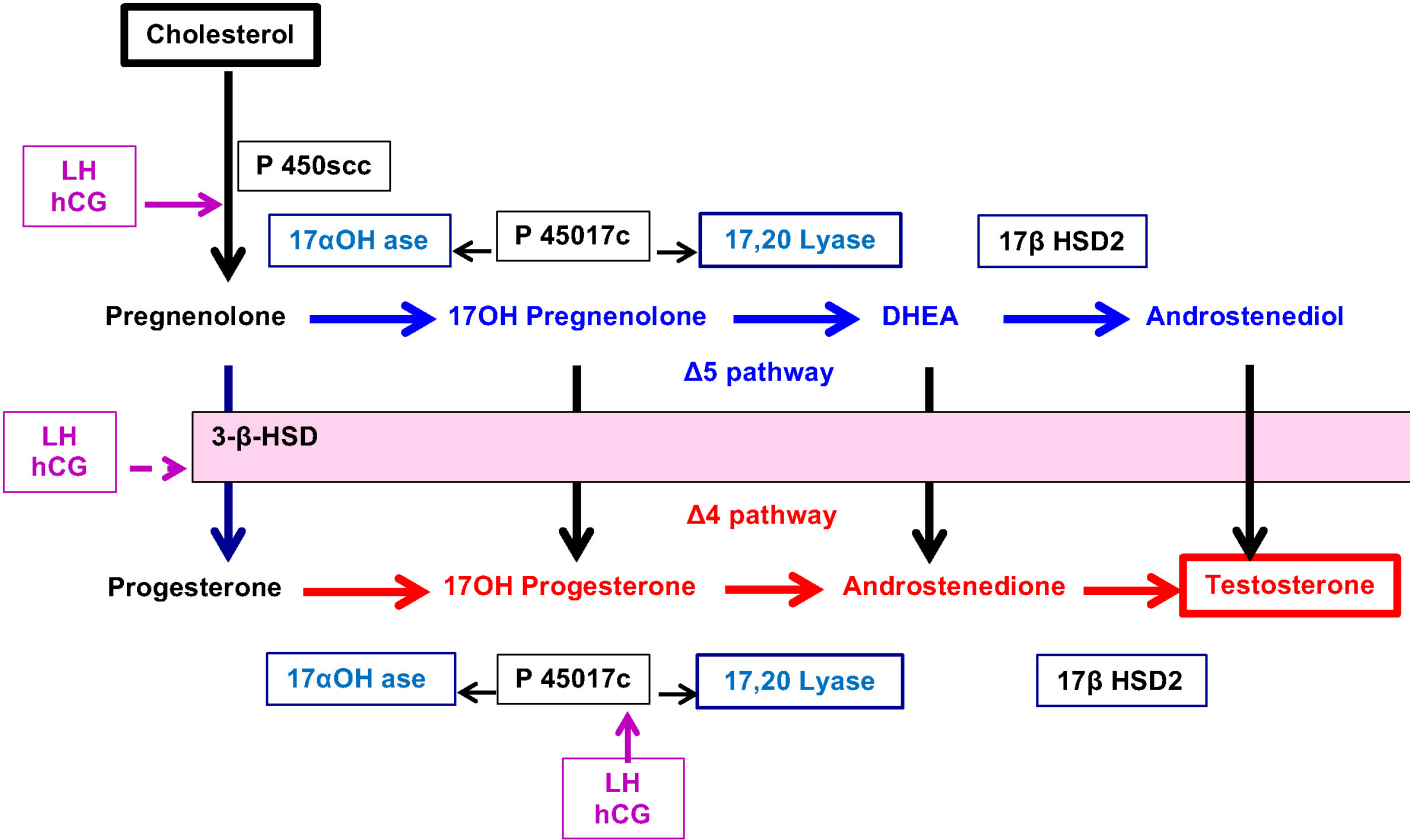
Schematic representation of enzymatic control of ovarian steroidogenesis.

Classically, a cell is called “steroidogenic” if it expresses the cholesterol side-chain cleavage enzyme, P450scc, which catalyses the first step in steroidogenesis. The conversion of Cholesterol to Pregnenolone in mitochondria is the first rate-limiting and hormonally regulated step in the synthesis of all steroid hormones. Therefore, P450scc is a key factor because it determines net steroidogenic capacity, so it serves as the chronic regulator of steroidogenesis. Ovarian expression of P450scc induced by LH-mediated cAMP, cleaves the 20,22 bond of insoluble cholesterol to produce soluble pregnenolone (**Figure 3**). Then, Pregnenolone may be metabolized into 2 distinct directions.

⚬ Pregnenolone may exit the mitochondria to become the substrate for P450c17 in the ER which catalyses both 17-hydroxylase and 17,20 lyase activities and produce 17OH-Pregnenolone and DHEA (Δ5 pathway).
⚬ Alternatively, Pregnenolone may be converted to Progesterone by 3ß-hydroxysteroid dehydrogenase (3ßHSD) present in mitochondria. This enzyme similarly controls, in the ER, the conversion of hormones from Δ5 pathway to Δ4 pathway: 17α-hydroxy-pregnenolone to 17α-hydroxyprogesterone (17OHP), DHEA to Androstenedione and Androstenediol to Testosterone.
⚬ In addition, P450c17 acts to convert Progesterone to 17OH- Progesterone and Androstenedione.
⚬ The final step leading to the production of Testosterone is controlled by 17ß Hydroxysteroid dehydrogenase (17ß HSD2).

Another key point in ovarian steroidogenesis is that granulosa cells do not express P450c17. Thus, in general, steroidogenesis is initiated in granulosa cells under the influence of LH which stimulates the expression of P450scc. Pregnenolone and Progesterone from granulosa cells diffuse into adjacent theca cells where they can be acted upon by P450c17 and 3ßHSD to produce Androstenedione which is partly converted to testosterone by 17ß HSD2. Finally, testosterone leaves the theca cell compartment to granulosa cells where it is converted to Oestradiol by aromatase (CYP) stimulated by FSH.

These data attest for a critical role of both cholesterol metabolism inside mitochondria to initiate steroidogenesis and steroidogenic enzymes of ovarian compartments to produce oestradiol and progesterone on one side (granulosa cells) and androgens on the other side (theca cells).

## Relationship between ovarian sensitivity to FSH and steroidogenesis

This very close relationship between androgen production and folliculogenesis was the rationale of the study conducted by Prof Okten’s team in Turkey (16). The main objective here was to perform an extended analysis of the steroidogenic capacity of human luteinized granulosa cells obtained in young patients who experimented hypo-response to ovarian stimulation related to poor ovarian reserve. For that purpose, 40 young (35 yrs) women programmed in an IVF program following stimulation by r.hFSH with GnRH antagonist were included in this study. Twenty of them were control patients who had documented a normal response to ovarian stimulation (8-15 retrieved oocytes) whereas the remaining 20 were classified as poor responders because of the collection of 3 oocytes in the current IVF cycle and a diminished ovarian reserve (DOR: AFC < 5 and AMH < 1.1 ng/ml).

The luteinized granulosa cells were obtained from follicular aspirates during the oocyte retrieval procedure and cultured individually. Their analysis allowed to get the following results:

- mRNA expressions of steroidogenic components (StAR-3ßHSD-aromatase) and of gonadotropin (FSH-LH) receptors measured by qRT-PCR were significantly decreased in poor responders as compared to control group. These data were confirmed by immunoblot analysis who showed that expression of StAR, 3ßHSD and aromatase was decreased in granulosa cells of poor responders.
- Basal steroidogenic capacity of granulosa cells, assessed by *in vitro* E2 and P production, was significantly reduced in poor responders. Furthermore, in accordance with a defective expression of gonadotropin receptors, granulosa cell response to FSH (upregulation of StAR and E2 production) and to hCG (increased expression of StAR and P production) were significantly lower in poor responders as compared to control group. The data attest for a significant deficit in basal/stimulated steroidogenesis in poor responders.
- The conventional immunofluorescence microscopy examination displayed a reduced cytoplasmic accumulation of intracellular lipids in the cells of poor responders. A more detailed analysis by confocal microscopy showed that the lipid content inside mitochondria was significantly decreased. Additionally, the confocal images analysis indicated that the signal intensities of StAR and 3ßHSD were significantly reduced and their colocalizations with mitochondria were markedly reduced.
- Finally, a substantial delay and reduction in cholesterol uptake, cytoplasmic accumulation and transportation to mitochondria were observed in poor responders. This reduced cholesterol trafficking was related to a decreased expression of LDL receptors.

Taken together, these findings, translated into clinical practice, indicate that POR to stimulation in young individuals with DOR should not simply be considered as a state of lesser follicle growth or oocyte yield. Rather, the underlying molecular perturbations of POR are much more complex and involve multiple steps of steroidogenesis (HDL receptors, cholesterol trafficking, expression of steroidogenic enzymes) and gonadotropin responsiveness.

To date, a limited number of studies have analysed the steroidogenic characteristics of luteinized GCs obtained from a heterogenous population of patients with DOR, advanced age, and POR. The existing data are somewhat inconsistent mainly because of methodologic or technical limitations (17, 18). In accordance with Turkish data, Phy et al. (19) demonstrated lower StAR expression and P4 production in the luteinized GCs of patients with DOR. In addition, it was previously reported that granulosa cells of elderly women (38-41 years) display defective mitochondria and fewer lipid droplets than the cells of a younger group (20, 21) and a reduced mitochondrial DNA content (22). Therefore, it might be speculated that ovarian hypo-response to stimulation of young women reflects an ovarian premature senescence. Although there is still no consensus on the exact definition of premature senescence, recent studies using whole-genome methylation array reported that luteinized granulosa cells of advanced-age women with DOR have a distinct epigenic profile and harbour a high frequency of epimutations suggestive of premature aging (23, 24).

Another issue to be addressed is whether the analysis of luteinized granulosa cells actually reflects the quality of ovarian steroidogenesis in absence of stimulation. In these young poor responders, theca cell function was not specifically assessed but, presumably, androgen (androstenedione, testosterone) biosynthesis is going to be reduced due to defective cholesterol trafficking inside mitochondria and low steroidogenic enzyme expression as well.

The close relationship between androgen production and folliculogenesis allows to assume that a hypo-androgen situation might participate in the event of ovarian hypo-response. Previous publications reported a significant decrease in androgen production in elderly women (25), a hypo-response to hCG (26) and a close correlation between basal androgen levels and the number of collected oocytes in normo-responders (27). These data are consistent with previous report (28) that, in young poor responders referred as group 3 in Poseidon classification, serum testosterone and androstenedione values were significantly lower than in the control group and in the Poseidon group 1. Nevertheless, as serum and follicular fluid testosterone concentrations are not positively correlated (29), androgen concentrations within follicular fluids were not significantly different (30).

## Therapeutic strategies in women classified as Poseidon Group 3

Altogether, these data indicate that the degree of androgen production is likely to be predictive of the granulosa response to FSH. Therefore, what benefit could be expected from improvement of androgen environment in poor responders? Furthermore, as androgens interfer from the early stages of folliculogenesis, any addition of drugs should be prescribed prior to the ovarian stimulation itself. Such a “priming effect” can be achieved throughout two different approaches:

*either a “systemic androgen priming” via supplementation with androgen (usually gel application) to increase circulating androgen levels.
*or an “intra-ovarian androgen priming” via administration of LH-like products and/or aromatase inhibitors to increase local androgen production both being performed during the period preceding the ovarian stimulation.

### Androgen supplementation

The first prospective, randomized clinical trial (31) indicated that a pre-treatment with daily application of testosterone gel (10 mg/day) during 15 days was associated with a significant increase in serum Testosterone values (from 0.58 ± 0.16 to 1.55 ± 0.89 ng/ml (P < 0.0001) but the improvement in ovarian parameters following this protocol was limited to a modest increase in the number of mature oocytes. Nevertheless, in accordance with other reports (32–35), a recent meta-analysis (36) of 8 randomized clinical trials (797 women) showed that a daily application of Testogel (10 to 12.5 mg/d) during 10 to 56 days before ovarian stimulation can significantly improve ovarian response to FSH stimulation:

* Increase in the number of cumulus-oocyte complex (WMD 0.88, 95% CI 0.22-1.54).
* Decrease in the duration of treatment & requirement of lower gonadotrophin doses (WMD - 368.8 IU, 95% CI - 612.4 to -125,3 IU).
* Higher clinical pregnancy and birth rates (RR 2.25, 95% CI 1.54-3.30 and RR 2.07, 95% CI 1.09-3.92 respectively).

Conversely, no differences were observed regarding the numbers of follicles ≥ 17mm, metaphase II oocytes, two-pronuclear oocytes and embryos transferred.

In addition, some limitations were underlined by the authors: a large variability in the definition of poor ovarian response (< 4 oocytes retrieved in previous stimulations despite high doses of gonadotrophins - Bologna criteria - Poseidon criteria), some concerns regarding the randomization process (only 3 studies at low risk) and, finally, a high heterogeneity regarding the duration of testosterone pre-treatment. Interestingly, the largest randomized clinical trial which compared 4 and 6 weeks of testosterone application could not find any difference between the 2 groups regarding the number of collected oocytes or IVF out-come (37). This conclusion was confirmed in another prospective study comparing the effects of 10 to 56 days of androgen gel application to a small number of poor responders (38). Even if a short supplementation may offer the advantages of lower cost, patient-friendly prescription and reduction in the risk of therapy interruption, these data do not support the concept that a long-term effect of androgens is required to improve folliculogenesis. Unfortunately, no data actually exist regarding the consequences of short or long-term systemic testosterone administration on intra-follicular androgen levels.

No side effects were reported whatever the duration of gel application. However, the long- term effects of testosterone pretreatment have not been currently studied. Similarly, no data are available on children born after testosterone pretreatment.

Altogether, these data indicate that systemic androgen supplementation is likely to be beneficial not only on stimulation parameters but, most importantly, in terms of live birth rate. Nevertheless, additional prospective randomized studies are needed in a large number of poor responders even if everybody knows that this population is rather reluctant to be included in randomized trial. In addition, relevant RCTs should allow to determine the optimal duration of supplementation in a well-defined population of poor responders. This therapeutic approach may offer new interesting perspectives in young women whose prognosis of fertility is likely to be fair because the embryonic aneuploidy rate is still low (39).

### Stimulation of androgen production (LH-hCG-aromatase inhibitors)

As Luteinizing Hormone (LH) plays a crucial role in folliculogenesis, several studies assessed the efficacy of LH supplementation in IVF protocols using GnRH agonists/antagonists in patients with normal ovarian function and some beneficial effects of LH addition were observed in elderly (> 35yrs old) women (40) and hypo-responders (4-9 oocytes) (41). In contrast, meta-analyses focused on women with poor response to FSH reported discordant conclusions (42, 43). In the specific group of young women with a poor response to FSH according to Poseidon classification, no clear advantage of adding LH to FSH could be demonstrated in Group 3 (44) as well as in Group 4 (45).

However, in these studies, FSH and LH were concomitantly administered during the stimulation period. Therefore, the potential benefit of an “intra-ovarian androgen priming” was not assessed except in few situations:

* In women with hypo gonadotrophic hypogonadism, a 7-day priming with 300 IU of LH allowed to reduce the FSH threshold (46).
* Another study performed in normo-responders included in GnRHa stimulation protocol showed that a 7-day administration of 300 IU/d LH during the period of hypophyseal desensitization was associated with a modest clinical benefit (47).
* Finally, a randomized clinical trial performed in < 38yrs old women with previous poor response to FSH (n=43) showed that a pre-treatment of 4 days with 150 IU LH was associated with a modest increase in the number of oocyte and live birth rate (48).

However, to the best of our knowledge, no randomized clinical trial has been yet planned to assess the interest of LH priming in young poor responders classified in Poseidon Group 3. Meanwhile, the recent work by Casarini’s team using human luteinized granulosa cells from normo (> 10 oocytes), hypo (4-9 oocytes) and poor responders (< 3 oocytes) (49) aimed to study the effects of *in vitro* LH addition on the FSH dose-response curves for cAMP and Progesterone production. Interestingly, this study showed that the efficacy of FSH is lower in hypo and poor than in normo-responders but addition of picomolar concentrations of LH increased the efficacy of FSH, bringing their response pattern like that of normo-responder cells treated with FSH alone.

Therefore, it is still uncertain whether LH supplementation of poor responders (Poseidon Group 3) could be effective to improve the ovarian response to FSH. As previously reported (50), the use of GnRH agonist test (GAST) with measurement of serum 17 OH Progesterone, a more sensitive index than Testosterone, could allow to identify patients who might benefit from addition of LH. Hopefully, these data could stimulate clinicians to launch new randomized prospective studies on that issue, even if the limitation of protocols with LH supplementation will be the cost effectiveness.

An alternative way to stimulate androgen production could be the use of hCG, another product with LH activity. Nevertheless, it should be emphasized that, while interacting with the same receptor, biological activity of LH and hCG are quite distinct. Indeed, recombinant LH exerts both proliferative and antiapoptotic actions through phosphorylated extracellular-regulated kinase 1/2 (pERK1/2) and phosphorylated AKT signals. By contrast, LH activity provided by hCG displays a steroidogenic and proapoptotic effect mediated by cAMP and protein kinase A (PKA) (51). Therefore, the specific effect of hCG priming needs to be specifically evaluated.

A limited number of studies assessing the effects of hCG prior to FSH administration has been performed up to now and a great majority associated the administration of aromatase inhibitors to block the conversion of androgen to oestrogen. Two preliminary studies were performed in women with normal ovarian function in order to assess the benefits of a “short-term androgen priming” using aromatase inhibitor and hCG administered during a early follicular phase down regulated by GnRH antagonist. The first one (52) tested the effects of a 7 day-period administration of aromatase inhibitor (Letrozole 2.5 mg/d) ± hCG (2.500 IU twice a week). A significant increase in follicular fluid levels of testosterone was observed in women receiving aromatase inhibitors, in accordance with previous reports (53) but most of the characteristics of ovarian stimulation and cycle outcome were unchanged. The second study published by the same Danish team (54) was prospective, randomized with a period of androgen priming limited to 3 days. Serum testosterone levels were slightly higher in the treated group but no significant increase in top quality embryos could be observed. These studies performed in women with normal ovarian function validated the concept of “short-term intra-ovarian androgen priming” using aromatase inhibitors and hCG to improve the androgen environment. Until now, very few studies have been performed in patients classified as poor responders according to Bologna criteria. The study by Bercaire et al. (55) assessed the effects of a combination of 3 drugs applied simultaneously during the cycle (28 days) preceding the FSH stimulation to poor responders: Transdermal testosterone 25 mg every other day, Letrozole 2,5 mg/d and hCG 2.500 IU twice a week. Unfortunately, this protocol was performed in a small number of < 40 yrs old women, preventing any firm conclusion. Further to previous observations from women with normal ovarian function, a “long-term intra-ovarian androgen priming” was proposed in order to boost the recruitment of small pre-antral follicles (56). For that purpose, during 2 months of hypophyseal desensitization induced by GnRH agonist (Depot formulation 3,75 mg every 4 weeks), a small dose of recombinant HCG (260 IU every second day) was administered subcutaneously in addition to oral letrozole 2.5 mg daily for 8 weeks. The results of this study were rather disappointing because the mean serum AMH levels decreased over time and no significant effects on Antral Follicular Count (AFC) could be observed. In addition, serum testosterone remained unchanged. Several reasons could explain the lack of benefit of this regimen: a too severe and prolonged pituitary down-regulation, an insufficient dose of hCG delivered every 2 days, a small number of patients (30), the single ethnicity population included. Consequently, another study was set up including women with low serum AMH who underwent 2 consecutive identical stimulation cycles separated by 8 weeks (2 cycles) of daily injection of 260 IU hCG (57). The primary end point was the assessment of FORT defined as the number of preovulatory follicles (>16mm) on hCG trigger day divided by the number of antral follicles (2–10 mm) at baseline who was similar in the 2 stimulation cycles. Surprisingly, circulating serum androgen levels were not increased at the end of the hCG priming period and testosterone levels within the follicular fluid of >17mm aspirated follicles were decreased at the end of the second cycle. Therefore, to validate the interest of a long-term hCG priming, the authors suggested to design a new study with the collection of follicular fluid from antral follicles before and immediately after priming in order to measure androgen levels and Androgen receptor and FSH receptor expression in small follicles (58). Additionally, these negative results should be interpreted in light of the data reported by Okten’s team (16). Indeed, the significant deficit in basal / stimulated ovarian steroidogenesis reported in young poor responders could definitely explained the absence of positive effect of hCG administration. If we translate these data in clinical practice, this might indicate that no significant improvement in ovarian response can be expected when hCG in combination with Aromatase Inhibitors are prescribed in Poseidon 3 poor responders.

## Conclusion

The actual involvement of androgens in the state of poor ovarian response to stimulation is highly suggested by several studies. However, additional research is needed in this area before clear recommendations about the value of systemic or intra-ovarian androgen priming in a clinical setting can be made. The recent study analysing the steroidogenic capacity of granulosa cells attests for a significant deficit in basal / stimulated steroidogenesis in poor responders. If these results are confirmed, they would lead to recommend a systemic rather than an intra-ovarian androgen priming. In addition, it is our feeling that a special focus should be made on young women with reduced ovarian function (Group 3 of Poseidon classification) because euploidy rate in their oocyte and embryos seems to be still preserved (39).

## Author contributions

The author confirms being the sole contributor of this work and has approved it for publication.
